# Non-invasive Eye Tracking Methods for New World and Old World Monkeys

**DOI:** 10.3389/fnbeh.2019.00039

**Published:** 2019-03-05

**Authors:** Amy M. Ryan, Sara M. Freeman, Takeshi Murai, Allison R. Lau, Michelle C. Palumbo, Casey E. Hogrefe, Karen L. Bales, Melissa D. Bauman

**Affiliations:** ^1^The UC Davis MIND Institute, University of California, Davis, Sacramento, CA, United States; ^2^Department of Psychiatry and Behavioral Sciences, University of California, Davis, Sacramento, CA, United States; ^3^California National Primate Research Center, University of California, Davis, Davis, CA, United States; ^4^Department of Psychology, University of California, Davis, Davis, CA, United States; ^5^Platform Technology Research Unit, Drug Research Division, Sumitomo Dainippon Pharma Co., Ltd., Osaka, Japan

**Keywords:** eye tracking, nonhuman primate, rhesus macaque, titi monkey, social neuroscience

## Abstract

Eye-tracking methods measure what humans and other animals visually attend to in the environment. In nonhuman primates, eye tracking can be used to test hypotheses about how primates process social information. This information can further our understanding of primate behavior as well as offer unique translational potential to explore causes of or treatments for altered social processing as seen in people with neurodevelopmental disorders such as autism spectrum disorder and schizophrenia. However, previous methods for collecting eye-tracking data in nonhuman primates required some form of head restraint, which limits the opportunities for research with respect to the number of or kinds of primates that can undergo an eye-tracking study. We developed a novel, noninvasive method for collecting eye tracking data that can be used both in animals that are difficult to restrain without sedation as well as animals that are of different ages and sizes as the box size can be adjusted. Using a transport box modified with a viewing window, we collected eye-tracking data in both New (*Callicebus cupreus*) and Old World monkeys (*Macaca mulatta*) across multiple developmental time points. These monkeys had the option to move around the box and avert their eyes from the screen, yet, they demonstrated a natural interest in viewing species-specific imagery with no previous habituation to the eye-tracking paradigm. Provided with opportunistic data from voluntary viewing of stimuli, we found that juveniles viewed stimuli more than other age groups, videos were viewed more than static photo imagery, and that monkeys increased their viewing time when presented with multiple eye tracking sessions. This noninvasive approach opens new opportunities to integrate eye-tracking studies into nonhuman primate research.

## Introduction

Many cognitive processes are involved in navigating the primate social world, from identifying others in the group, evaluating social situations, and using visual communication to develop social relationships. How the primate brain interprets the social world through the visual system is critical from a social neuroscience perspective, and researchers have begun studying the complex processes which underlie social cognition, such as attention, face processing, and emotion recognition (Chang et al., 2013). The use of eye-tracking technology has allowed researchers to quantitatively evaluate an animal’s visual attention—the initial process that directs information to the brain.

Eye-tracking technology measures visual attention by quantifying various features of eye movements such as changing patterns of gaze direction and fixation durations. Because eye tracking measures natural visual responses of a participant, eye-tracking methods can be applied to humans, including very young infants, and individuals with neurodevelopmental and other disorders (Venker and Kover, 2015), as well as nonhuman animals (Machado and Nelson, 2011). For this reason, eye tracking makes it possible to test hypotheses in humans and nonhuman animals with similar methods, which provides a strong translational opportunity for the study of human cognition with animal models, especially nonhuman primates (Phillips et al., 2014).

Eye-tracking is used in nonclinical populations of humans to address a wide range of questions from social neuroscience (Birmingham and Kingstone, 2009) to marketing and consumer research (Khachatryan et al., 2017) and user experience with technology (Ioannidou et al., 2017). Using eye tracking in typically developing humans, researchers have demonstrated that people preferentially attend to eyes when viewing social stimuli (see Birmingham and Kingstone, 2009 for review) and process faces holistically rather than by their cumulative parts (Williams and Henderson, 2007). Eye-tracking also affords opportunities to understand attention in preverbal infants and has shown that eyes capture infants’ attention more so than other parts of the face (Di Giorgio et al., 2013) except that gaze shifts to the mouth region when infants are learning speech (Lewkowicz and Hansen-Tift, 2012).

Eye-tracking methods have also increased our understanding of how nonhuman primates process social information. Rhesus macaques (*Macaca mulatta*) can process two-dimensional face stimuli as faces (Sliwa et al., 2011) and even respond differently to stimuli of familiar faces as compared with unfamiliar ones (Gothard et al., 2004; Landi and Freiwald, 2017). Like humans, macaques focus more on the eye region than other parts of the face (Gothard et al., 2004; Dahl et al., 2009) and utilize information gained through gaze detection (Leonard et al., 2012; Putnam et al., 2016). Although the majority of nonhuman eye tracking studies have utilized macaques, a recent cross species comparison of humans, great apes and macaques reveals individual- and species-specific viewing patterns for social stimuli (Kano et al., 2018). Collectively, these studies in both humans and nonhuman primates provide insight into how primates navigate their social worlds and the neural substrates that support these functions.

In humans, studies about face processing that used eye tracking methods have yielded unique insights into how neurodevelopmental disorders such as autism spectrum disorder (ASD) and schizophrenia (SZ) can affect the processing of social information. People with ASD consistently show atypical gaze patterns with social stimuli such as reduced gaze directed at the eye region of faces and increased attention to nonsocial components of stimuli as opposed to social ones (reviewed by Papagiannopoulou et al., 2014; Black et al., 2017; Frazier et al., 2017). Likewise, individuals with SZ also demonstrate face processing deficits and exhibit a “restricted” strategy in their visual attention with shorter scanpaths (Loughland et al., 2002; Marwick and Hall, 2008). Our laboratory has utilized eye tracking to evaluate social development in nonhuman primate models of neurodevelopmental disorders (Bauman et al., 2014), and have found that rhesus macaques born to dams that experienced immune activation during pregnancy demonstrate face processing deficits that parallel findings from humans with both ASD and SZ (Machado et al., 2015). These results demonstrate how the ability to use eye-tracking methodologies to test hypotheses in humans and nonhuman primate models alike is important for our understanding of neurodevelopmental disorders (Millan and Bales, 2013).

However, regardless of the specific type of eye-tracking methodology used, the participant’s eyes need to be calibrated with the gaze-tracking technology. In previous studies, eye tracking in nonhuman primates has largely entailed more invasive approaches that require some form of head restraint to accurately calibrate and collect data. These invasive approaches increase the cost of research and decrease the number of individuals tested in eye-tracking studies. The traditional way to measure eye movement in nonhuman primates has been via a scleral magnetic search coil surgically implanted in the sclera of eye (Judge et al., 1980). Yet, recent improvements in computer software have allowed for optical-based tracking of eye movement, in which video-based trackers measure the orientation of the eye via corneal reflection of an infrared light source relative to the pupil center of the eye (Duchowski, 2017). While optical tracking software removes the need for surgical installation of the scleral coil, the head of the research subject has to be still so that the tracker does not mistake head movements for eye movements. For nonhuman primates, it is a common practice to prevent movement through the use of head posts. During the eye-tracking procedure, the head post is affixed to another apparatus such as a primate chair in order to keep the head stationary during eye-tracking (Gothard et al., 2004; Gothard et al., 2009; Mosher et al., 2011; Mitchell et al., 2014; Roy et al., 2014). While head restraint may continue to be necessary for some eye-tracking paradigms, such as those that include neurophysiology, researchers have sought to create more naturalistic eye-tracking methods in which the eye-tracker is able to collect accurate eye-tracking data while the monkey’s head is less restrained.

Less invasive eye-tracking methods benefit an animal’s welfare more so than invasive ones and with respect to translational aims, less invasive approaches more closely approximate human eye tracking testing settings. Apes, for example, have been trained to enter a testing arena voluntarily and drink from a juice dispenser while viewing stimuli projected from an apparatus on the other side of a clear acrylic divider (chimpanzees: Kano and Tomonaga, 2009; gorillas: Kano et al., 2012; orangutans: Kano et al., 2012; Pritsch et al., 2017) or participate in eye-tracking studies in their home cage with no prior training or juice dispenser needed (Howard et al., 2017). For rhesus macaques, infants have been tested in eye-tracking paradigms while remaining in ventral contact with their mother, who was lightly sedated and placed in a reclining chair during the test (Muschinski et al., 2016). Noninvasive ways to stabilize the head have also been devised for older rhesus macaques both for eye-tracking paradigms and other studies such as for neural recordings. Researchers have created thermoplastic masks that hold the monkey’s head in place via attachments to a primate chair (Machado and Nelson, 2011; De Luna et al., 2014; Amemori et al., 2015; Drucker et al., 2015; Machado et al., 2015) or transport box (Fairhall et al., 2006). This method eliminates the need for surgery, although a light sedation may still be used prior to the eye-tracking session in order to mold the mask to the shape of the monkey head for a secure fit (Machado and Nelson, 2011; Machado et al., 2011; Amemori et al., 2015; Drucker et al., 2015; Machado et al., 2015). Studies that use eye-tracking methods in freely-moving adult monkeys without masks or sedation have been conducted previously with boxes that have a small viewing area, although training has been required for the monkey to acclimate to the experimental procedure (Bagshaw et al., 1970; Wilson et al., 2010). More recently, eye-tracking data were successfully collected from capuchins that have been previously trained to enter a testing cubicle where they viewed stimuli through a mesh screen (Howard et al., 2018).

In order to extend the current use of noninvasive approaches to eye tracking in nonhuman primates, we carried out a pilot study to evaluate feasibility of using noninvasive eye tracking approaches in two species commonly studied in behavioral neuroscience, the rhesus macaque and the monogamous coppery titi monkey (*Callicebus cupreus*). Because we were testing different species and individuals at multiple developmental time points, we sought to use streamlined methods for collecting eye-tracking data that did not require previous surgeries, sedation events, restraint during data collection, or previous training to acclimate to the testing procedure. Below, we describe a novel approach to measuring eye tracking in unrestrained monkeys with no habituation, which was effective in both New and Old World monkey species and across different ages and sizes in infant, juvenile, and adult monkeys.

## Materials and Methods

We developed our experimental procedures in collaboration with veterinary, animal husbandry, and behavioral health staff at the California National Primate Research Center (CNPRC). Our protocols were approved by the University of California, Davis Institutional Animal Care and Use Committee. All attempts were made to promote the psychological well-being of the animals that participated in this research via social housing, enriched diet, use of positive reinforcement strategies, and minimized duration of daily testing sessions.

### Subjects and Living Conditions

#### Rhesus Macaques

We used six infant (one male, five females) and four juvenile (two males, two females) rhesus macaques from the CNPRC colony. Infants were housed indoors with their dams in standard laboratory caging and were enrolled in the eye-tracking study from 1 to 4 months of age. Juveniles were raised by their dams in large field cage enclosures before moving indoors at approximately 1 year of age where they were continuously pair housed in same-sex pairs and were approximately 2 years and 4 months old at the time of testing. During this time, the four juveniles participated in twice weekly socialization in a large, group-housing environment (4.3 × 1.5 × 2.1 m). Housing for both infants and juveniles were maintained on a 12-h light/dark cycle and were continually monitored for temperature and humidity. Macaques were fed monkey chow, biweekly fresh produce, and water was available *ad libitum*.

#### Titi Monkeys

Subjects included eight juvenile (three males, five females) and 11 adult (six males, five females) laboratory-born titi monkeys from the CNPRC colony. Adult titi monkeys at the CNPRC live as heterosexual pairs in stable family units with juvenile and infant offspring, if relevant. Adult subjects in our study were either paired and living with their pair mate or were unpaired (awaiting a pair mate) and living with a juvenile offspring. Juvenile monkeys were living with their parent(s). Titi monkeys were housed in cages (1.2 × 1.2 × 2.1 m) that are situated so that each family group was visually isolated from others, but auditory and olfactory interactions were possible. Animals were on a 12-h light/dark cycle and temperature was maintained at 21°C. Animals were fed a diet of monkey chow, rice cereal, apple, raisins, banana, and carrot. Water was available *ad libitum*.

### Interpupillary Distance

One of the primary concerns for collecting noninvasive and unrestrained eye-tracking data from titi monkeys and young rhesus macaques was that their interpupillary distance, or the distance between their two pupils, may be too small for an eye tracker to detect and distinguish both eyes. In order to evaluate the potentially lower limit of interpupillary distance that the eye-tracker system could detect, we opportunistically measured the interpupillary distance of both species. The distances are presented in Table 1 with averages calculated when more than one monkey was sampled.

**Table 1:** Interpupillary distances of rhesus macaques and titi monkeys.

Species	Life stage	Interpupillary distance
Rhesus Macaque (*Macaca mulatta*)	Infant (1 week old)	22 mm (*n* = 1)
	1 month old	22 mm (*n* = 1)
Titi Monkey (*Callicebus cupreus*)	Juvenile	15 mm (*n* = 3)
	Adult	19 mm (*n* = 3)

### Noninvasive Eye Tracking

We used a Tobii Pro TX300 eye tracker with a sampling rate of 120 Hz and Tobii Studio optical tracking software to record and process noninvasive eye-tracking data (Tobii Technology, Stockholm, Sweden). The hardware and software were not modified from their default settings intended for human participants, including a fixation filter with a maximum gap length of 75 ms. The monkeys were also positioned at a distance from the eye tracker that is specified for humans by the manufacturer. At this distance (∼60 cm), the spatial resolution of the eye tracking system with respect to how precisely it can determine the location of a gaze is 0.06° for one eye and 0.04° for both eyes, which represents the gaze angle between the eye(s) and the screen.

#### Rhesus Macaques

**Figure 1:**
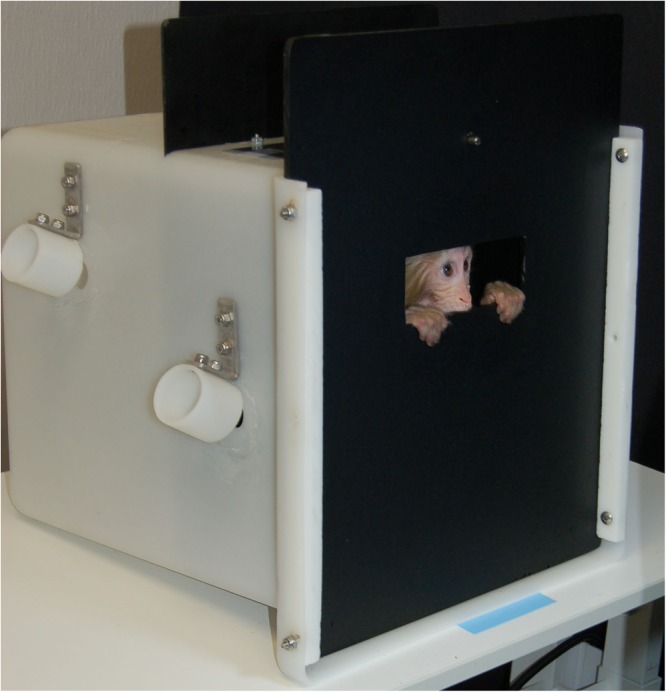
The modified transport box used with infant rhesus macaques.

Three methods for collecting infant eye-tracking data in rhesus macaques were evaluated. First, we used hand restraint with rhesus infants. After removal from the dam, the infant was transferred to a testing room using a transfer box (37.0 × 36.0 × 35.0 cm). An experienced researcher sat on a chair placed 55 cm in front of the eye tracker’s monitor display (1920 × 1080 pixels) and held the infant monkey swaddled in a towel. This method has been successfully used in surrogate-reared infant rhesus macaques (Paukner et al., 2013) and 6-month-old maternally-reared rhesus macaques (Alvarado et al., 2017). Our second approach with rhesus infants was with ventral contact with the dam. as reported by Muschinski et al. (2016), the dam was lightly sedated and the pair was transferred to the testing room. They were placed on a table in front of the monitor display with the dam in dorsal recumbancy and the infant in ventral contact with its mother and within view of the display. Finally, we used a modified transfer box (Figure 1). The infant was transported to the testing room in a transfer box following removal from the dam. In the testing room, the infant was placed in the modified transfer box with the same overall dimensions as the original box but with interior partitions added to reduce the amount of free space (14.0 × 14.0 × 35.0 cm). The front panel of the box was constructed of opaque black acrylic with a viewing window (4.5 × 12.5 cm) at approximately eye level. Subjects could still move freely in the compartment, however, the partitions and the window helped to direct gaze toward the eye tracker and display monitor. The box was placed 55–60 cm from the eye tracking monitor.

We ultimately selected the modified transfer box method for use in our subjects (Figure 1). Despite previous successes with hand-restraint (Paukner et al., 2013; Alvarado et al., 2017) and maintained ventral contact with the sedated dam (Muschinski et al., 2016), we determined following pilot testing that these methods would not be feasible for measuring eye gaze in our subjects. All reported eye-tracking data presented here were thus collected from monkeys contained in the modified transfer box.

For the juvenile macaques, a similar, but larger, enclosure was used for the eye-tracking test sessions. Juveniles were transferred from their home cage to the testing room by directly using the modified metal transfer box (31.0 × 34.5 × 40.0 cm). The box had a sliding door on one side and a fixed opaque black acrylic panel with a viewing window (15.5 × 5.0 cm) on the opposite side. The box was placed 55–60 cm from a monitor display with the viewing window oriented toward the display.

#### Titi Monkeys

**Figure 2:**
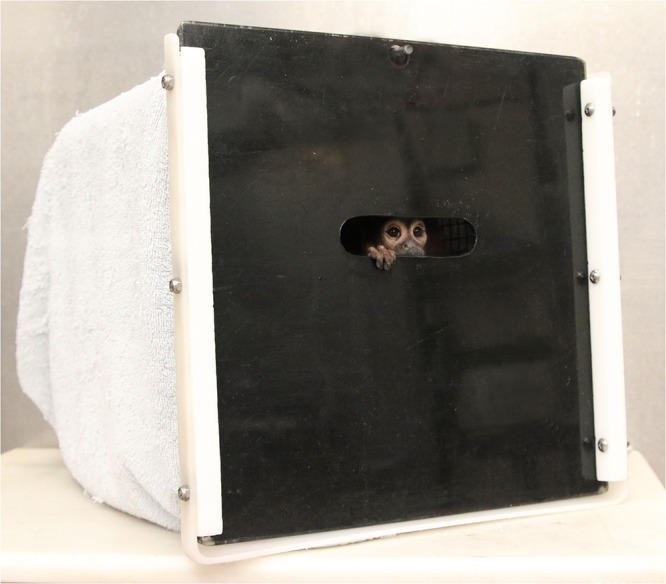
The modified transport box used for juvenile and adult titi monkeys.

The eye-tracking set up for the titi monkeys was similar to the approach described above for the infant and juvenile macaques. The titi monkeys were already trained to jump directly into a familiar transport box (approximately 31 × 31 × 33 cm), which was large enough to allow the animal to sit normally and turn around (Figure 2). They were also acclimated to sit quietly in these transport boxes for 30–60 min periods. Similar to the third approach described above for the rhesus subjects, we modified the door panel for the titi transport boxes so that it was black and had a small viewing window at eye level (8.0 × 3.0 cm) through which the titis could view the eye tracking stimuli on the display monitor. The titi monkeys were brought to the testing room in this modified transport box, which was placed 55–60 cm away from the eye-tracking monitor and covered with a towel to reduce visual access to any location other than the viewing window.

### Eye Tracking Procedure

The testing box containing the subject was placed 55–60 cm in front of the eye tracking monitor display (1920 × 1080 pixels). Overhead room lights were turned off and a single lamp provided illumination of approximately 300 lux around the viewing window. Black curtains enclosed the eye tracking test space to reduce environmental distractions.

### Rhesus Macaques

#### Calibration

Prior to data collection for each animal and session, we used a preset 5-point calibration procedure in Tobii Studio that is designed for use in human infants (Papageorgiou et al., 2014; Imafuku et al., 2017) and has been used previously in studies with rhesus macaques (Paukner et al., 2013; Simpson et al., 2017). Attention-grabbing calibration stimuli included a still image (fear grimace), video clips depicting rhesus monkey behavior (such as grooming, foraging, aggression, and play), and animated shapes. The calibration stimuli were used only for calibration and not repeated during data collection. A stimulus was chosen for calibration, and gaze data were collected when the animal looked at each of the five calibration points (four corners of the screen and then center) in succession on the screen. We assessed the success of a calibration based on the Tobii-generated error vectors for each of the five points. To get sufficient calibration data, we repeated calibration as needed and used the calibration session with the least amount of error before moving on to data collection. Stimuli presentation and data collection commenced immediately after the calibration.

#### Test Session

Following calibration, we presented four stimulus sets to the monkeys. Each monkey performed six eye tracking sessions within 60 days with a minimum of 3 days between sessions. The stimulus sets were presented in a random order within and between sessions. Each testing session, from calibration through data collection, did not exceed 60 min. Monkeys were fed sliced grapes before and after the test session.

In order to evaluate the feasibility of collecting eye-tracking data in our minimally invasive approach, we presented the monkeys with three stimulus sets that had previously been used in eye-tracking studies with rhesus macaques. Two stimulus sets were donated by Dr. Machado. One of these stimulus sets was of color photographs of macaque facial expressions (Machado et al., 2015) and the other consisted of video stimuli of monkey social scenes and nature scenes of landscapes, land mammals, marine mammals, birds, or insects or other invertebrates (Machado et al., 2011). The third stimulus set, donated by Dr. Paukner, consisted of primate social stimuli videos presented side-by-side with an abstract shape that continuously moved across the screen. A fourth stimulus of a mother–infant interaction video clip was also presented in order to increase the variety of stimulus sets presented to the rhesus macaques. More information on each stimulus set is provided in Supplemental Methods.

### Titi Monkey

#### Calibration

As with the rhesus macaques, we first used a 5-point calibration procedure with the titi monkeys prior to data collection in each session. Calibration stimuli included titi monkey videos with vocalizations included as well as animated shapes.

#### Test Session

We presented the titi monkeys with two stimulus sets. Each monkey participated in one eye-tracking session. The testing session, from calibration through data collection, did not exceed 30 min.

We are unaware of previous eye-tracking studies carried out in titi monkeys. For our stimulus sets, we therefore developed our own stimulus sets from archived photos and videos of titi monkeys from the CNPRC colony, with more details provided in Supplemental Methods. We used stimuli that had a variety of arrangements of animals, including solo and group images of adults, infants, and family groups as well as non-social images of caging and food.

### Data Analysis

We measured the success of our eye-tracking method by determining how well the eye-tracker was able to track the eyes of the monkeys and how often the monkeys looked at the presented stimuli. Tobii software provided a sampling percent for the eye-tracking session, which represented the number of eye-tracking samples that were correctly tracked or identified divided by the number of attempts by the system. However, the Tobii-generated sampling percent accounted for attempts at tracking eyes both at times when a stimulus was presented on the screen and the inter-trial-interval (black screen) between stimulus presentations (Tobii technical support, personal communication). We thus used total fixation duration as a more conservative measure of looking time than sampling percent, because total fixation only measured eye gaze during stimuli presentations. Fixations occur when the eye is still and when visual information is gathered, as opposed to saccades, which are eye movements. Total fixation duration in Tobii Studio is the sum of the duration of all fixations on the computer screen.

We summed the total fixation durations for each stimulus set that monkeys viewed in a testing session. In order to determine the overall percentage of time that the monkeys viewed stimuli, we calculated a percentage based on the sum of the total fixation durations divided by the number of seconds that stimuli were presented on the screen. Because the stimulus sets varied in total length of time presented, we used percentages to compare fixation time between stimulus sets. Finally, we calculated averages for the sampling percent and total fixation duration for each stimulus set species and developmental time point. We used mixed design ANOVAs to test for differences between developmental time points, sex, types of stimuli, and evaluate any potential interactions using an alpha of 0.05.

## Results

### Fixations and Sampling During First Eye Tracking Session

All 10 rhesus macaques (*N* = 6 infant and *N* = 4 juveniles) successfully calibrated and were tested on the stimulus sets for data collection. Five of the 19 titi monkeys (two juveniles, three adults) failed to calibrate and did not generate eye-tracking data for further analyses; all titi results are from the remaining 14 individuals. With no previous exposure to the eye-tracking system, in the first data collection session for 10 rhesus macaques and 14 titi monkeys, the eye-tracking computer successfully collected an average of 31.48% (±8.45 SD) and 29.14% (±15.0 SD) of its attempts at tracking rhesus macaque and titi monkey eyes, respectively. In the first session, rhesus macaques fixated on the screen during all stimuli presentations for an average of 156.83 (±46.05 SD) seconds which was 22.60% (±0.06 SD) of the possible fixation time on stimuli, and titi monkeys fixated on the screen for 44.65 (±22.24 SD) seconds which was 26.26% (±0.13 SD) of the possible fixation time on stimuli.

#### Age and Sex Effects

**Figure 3:**
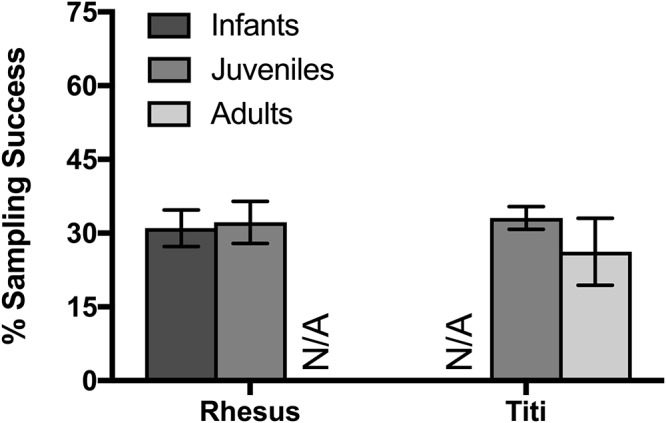
Effect of age on rhesus and titi monkey looking time, as measured by eye tracker success at detecting the eyes. Mean ± SEM.

To assess age and sex differences in eye-tracking success, we collected data from infant rhesus macaques, juveniles from both species, and adult titi monkeys. There were not a sufficient number of male macaques studied in order to test for sex differences in rhesus macaques, thus sex effects are only presented for titi monkeys. For both rhesus macaques in their first session and for titi monkeys, there was no significant difference in the sampling success from the eye-tracking system between the two age groups compared (rhesus macaque infant: 31.0% ± 0.09 SD, juvenile: 32.2% ± 0.09 SD; *t*(8) = 0.21, *p* = 0.842; titi monkey juvenile: 33.1% ± 0.02 SD, adults: 26.2% ± 0.19 SD; *t*(12) = 0.842, *p* = 0.416; Figure 3). Additionally, there was no difference in sampling success observed between male and female titi monkeys (males: 33.1% ± 17.03 SD, females: 26.2% ± 13.70 SD; *t*(12) = 0.842, *p* = 0.416).

**Figure 4:**
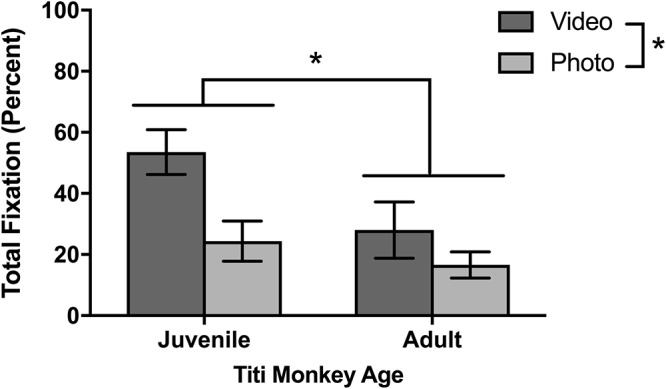
Effect of age and stimulus type on titi monkey looking time. Mean ± SEM. ^∗^*p* < 0.05.

**Figure 5:**
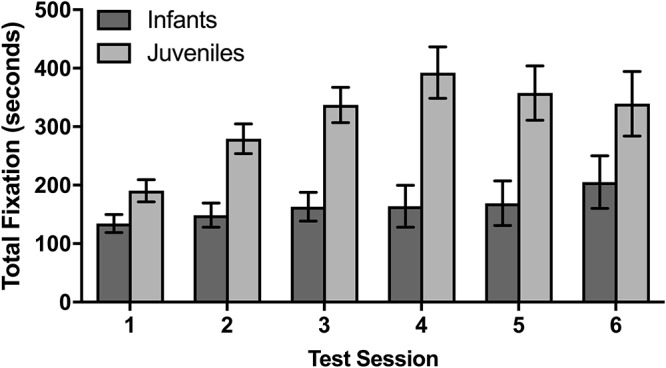
Effect of age and test session on rhesus macaque looking time. Mean ± SEM.

As for looking behavior measured by total fixation duration, juvenile titi monkeys fixated longer on the screen when stimuli were presented than adult titi monkeys [*F*(1,10) = 5.01, *p* = 0.048; Figure 4]. Yet, as with sampling success, there were no statistically significant differences between males and females in total fixation duration [*F*(1,10) = 0.007, *p* = 0.933]. For the rhesus macaques’ initial eye-tracking session, there was no significant difference in fixation duration between juvenile and infant rhesus macaques [*F*(1,8) = 0.021, *p* = 0.888; Figure 5], although this changed over the course of multiple testing sessions (see below).

### Longitudinal Eye Tracking (Rhesus Only)

The rhesus macaque infants and juveniles had six data collection sessions on 6 different days over the course of 2 months. In a mixed design ANOVA evaluating whether there were age effects on the total fixation duration across sessions, juveniles fixated significantly longer over the course of the six sessions than infants [*F*(1,8) = 18.31, *p* = 0.003]. There was also a significant difference between sessions [*F*(5,40) = 5.25, *p* = 0.001], with a planned comparison demonstrating that the average total fixation duration for the subsequent second through sixth sessions was significantly longer than the duration observed in the first session [*t*(54) = 2.13, *p* = 0.038]. There was a trend for an interaction between fixation duration across sessions and age [*F*(5,40) = 2.35, *p* = 0.058] which suggests that the monkeys at different developmental time points fixated differently across the multiple sessions, although this trend does not meet our statistical criteria for conducting subsequent post-hoc analyses. As there were no significant differences between age groups in the first session, these data suggest that the juveniles increased their fixation time after the first session more so than the infants.

### Photo Versus Video Stimuli

Although different stimulus sets were used for the two species, both the titi monkeys and rhesus macaques were presented with photo and video stimuli allowing for a broad comparison of these two categories. Titi monkeys fixated significantly longer on the video stimuli than on the photo stimuli [*F*(1,10) = 7.75, *p* = 0.019; Figure 4]. There were no significant interactions between age groups [*F*(1,10) = 1.49, *p* = 0.251] or sex [*F*(1,10) = 0.041, *p* = 0.844] with respect to age or sex groups fixating differently on photos or videos when compared with the other group.

**Figure 6:**
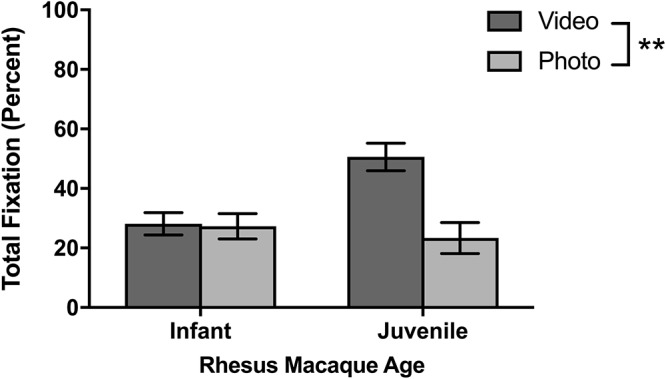
Effect of age and stimulus type on rhesus monkey looking time. Mean ± SEM. ^∗∗^*p* < 0.01.

The rhesus macaques viewed one photo stimulus set called “Facial Expressions” and three video stimulus sets of “Social vs. Nature,” “Social vs. Abstract,” and “Mother-Infant Interaction.” In the first session, there were no significant differences in fixations between the photo and video stimuli [*F*(1,8) = 0.815, *p* = 0.393] and no age differences or interactions between age and stimuli. For the average for all six sessions (Figure 6), the macaques fixated significantly longer on video than photo stimuli [*F*(1,8) = 20.14, *p* = 0.002]. There was a significant age by stimulus interaction [*F*(1,8) = 17.60, *p* = 0.003], as infants looked similarly across both kinds of stimuli [*t*(5) = 0.32, *p* = 0.765] yet juveniles fixated significantly longer on videos than photos [*t*(3) = 4.21, *p* = 0.024].

### Next Steps for Analysis

**Figure 7:**
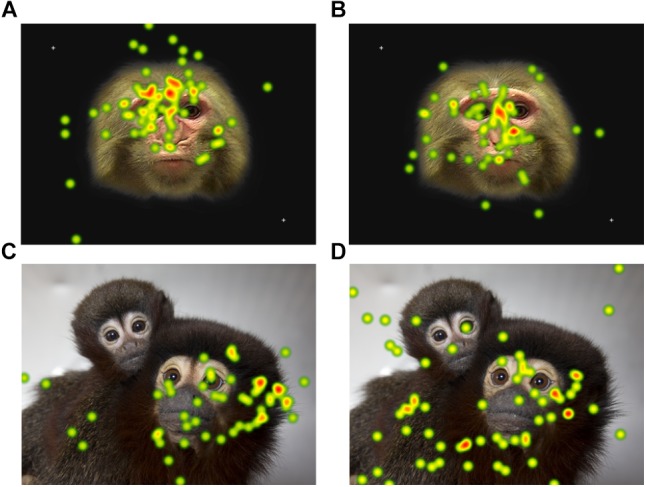
A heat map plot of combined fixation data for all participants for one of the static photo stimuli presented. The color of the plot becomes warmer (yellow and then red) as the number of fixations on the area increase. The rhesus macaque photos were presented for 15 s and the titi monkey photos for 10 s. The rhesus macaque data are for the first session only. Heat maps are presented separately for **(A)** infant rhesus macaques **(B)** juvenile rhesus macaques **(C)** adult titi monkeys **(D)** juvenile titi monkeys.

Provided with the opportunity to collect eye-tracking data from unrestrained monkeys, the next steps of the analysis will be to understand what parts of stimuli the titi and rhesus monkeys look at given that they can choose not to look at the stimuli or screen. For example, Tobii Studio software can generate heat map plots based on the number of fixations over the entire presented screen across all participants in the selected group. For both titi monkeys and rhesus macaques, the heat maps suggest that there are patterns for where the monkeys looked in our study based on the context of stimulus (Figure 7). Tobii Studio software can also generate fixation or gaze data for specific areas of interest the user specifies, even for such components as faces moving in a video presentation. We advise that the user consider the error vectors presented during calibration when considering the size of the areas of interest, for narrowly defined areas of interest such as strict borders around eyes may miss data from monkeys that fixated on the eyes but the eye-tracker calibration, while successful, places their fixation close to but not directly on the eyes in the stimulus.

## Discussion

Here we demonstrate novel methods for collecting eye-tracking data from rhesus macaques and titi monkeys across multiple developmental time points using a modified transport box to facilitate a noninvasive approach. Our results indicate that it is possible to successfully acquire eye-tracking data, even for monkeys that were not previously habituated to the modified transport box, eye-tracking room, or stimulus presentation. Moreover, this study provides benchmarks for eye tracking data using a variety of social, non-social, animated, and still stimulus sets. These methods advance eye-tracking opportunities for rhesus monkeys and establish feasibility of collecting eye-tracking data from the monogamous coppery titi monkey.

Previous eye-tracking studies with rhesus macaques align with findings from both human and ape studies that these primates prefer to view social stimuli, especially faces (macaques: Keating and Keating, 1982; Nahm et al., 1997; Gothard et al., 2004; Mosher et al., 2011; chimpanzees: Kano and Tomonaga, 2009; Hirata et al., 2010, humans: Goren et al., 1975; Farah et al., 1998). Across the primate order, social stimuli like a conspecific’s face may be a particularly salient stimulus, and one that the primate brain is particularly drawn to attending to in order to process and extract information from it. Even in the absence of reinforcement, macaques will view social stimuli in an eye-tracking paradigm (Mosher et al., 2011; Paukner et al., 2013). The results of our study align with this suggestion that macaques may have a naturally occurring interest in viewing social stimuli. The monkeys were unrestrained and thus able to turn around or divert their gaze from the stimuli, yet they nonetheless viewed 22.60% of the stimuli presentation during our data collection session. Furthermore, titi monkeys responded in a similar way and voluntarily viewed 26.26% of the stimuli presented in the absence of reinforcement and with opportunities to divert their eyes or face from the stimulus screen. As this was the first known eye-tracking study in titi monkeys, the data suggest that they have a similar naturalistic interest in social stimuli as other species of primates. Unfortunately, there is a not sufficient standardization of procedures for monkey eye-tracking studies in order to compare looking times across studies quantitatively, such as comparing the preference for social stimuli across studies. Methodological differences of prior training, head restraint, and food reinforcement can affect a monkey’s proclivity to look toward the screen. Here we provide a baseline for how monkeys view social stimuli in an eye-tracking paradigm in the absence of training, habituation, and restraint as a resource for future studies.

In addition to our results that macaques and titi monkeys will view stimuli voluntarily without previous habituation, our data suggest possible trends for opportunistically collecting eye-tracking data from monkeys using methods that are both noninvasive and do not use restraint. First of all, we found that rhesus macaques that were considered in the juvenile stage of their developmental trajectory fixated more on stimuli as a group than infants, although multiple sessions are needed to observe this effect in rhesus macaques. It is possible that the juvenile stage is the optimal developmental time point to collect eye-tracking data when compared with infant. However, with the small number of monkeys tested, it is possible that this relationship is driven by individual differences in fixation tendency between monkeys rather than differences based on developmental trajectory.

Secondly, both titi monkeys and rhesus macaques fixated more on video stimuli than photo stimuli, which may suggest that monkeys attend more to dynamic or moving stimuli rather than static images. Yet, there may be species differences in what monkeys attend to, and more kinds of stimulus sets would help to clarify whether there are other parts of the stimuli that are more important than movement (Kano et al., 2018). For example, the rhesus macaque static stimuli were of frontally oriented macaque faces that appeared to stare directly at the macaque subject. Machado et al. (2015) found that macaques had significantly higher measures of sympathetic arousal when they viewed videos of macaques that directed facial expressions toward them than when the subject monkey viewed videos of monkeys interacting with each other. Furthermore, when placed in more restrictive configurations for eye tracking such as a chair, it has been noted that rhesus macaques initially respond to static facial stimuli with lipsmacks, barks, and ear movements, although the macaques habituate to these stimuli over time (Gothard et al., 2004). As the macaques in our eye tracking paradigm could choose whether to view stimuli, it is possible that the prolonged direct eye contact was arousing, and our macaques may have averted their eyes from looking at these faces in lieu of showing a lack of interest in attending to static faces.

The rhesus macaques also had six eye-tracking sessions, in which the same stimuli were presented in a different order from the previous session. Both infants and juveniles increased their fixation duration in sessions following the first session, although the effect was stronger in juveniles than in infant macaques. These data suggest that the macaques did not habituate to the repeated presentation of stimuli. Instead, while the monkeys attended to stimuli in the first session with no prior habituation, it is possible that monkeys will increase their fixation time after the first session as they become familiar with the overall eye-tracking procedure. Testing a monkey on multiple eye-tracking sessions may thus be helpful in order to extract as much visual attention information as possible.

While we were successful in collecting opportunistic eye tracking data from monkeys in our modified transport box, there are some observations from our experience that may provide some future directions in order to improve the reliability and quality of eye tracking data collection. First of all, calibration was not always successful with our smaller monkeys, as five titis (two juveniles and three adults) failed to calibrate. While we were able to calibrate our infant rhesus macaques, we have experienced challenges in calibrating young rhesus monkeys in ongoing research projects. The Tobii software offers potential strategies to overcome a failed calibration, such as using a successful calibration from a similar monkey. However, we selected to only use calibrations for the individual in the moments before data collection.

Secondly, experimenters were in the room while eye-tracking data were collected and were able to view the monkey from the perspective of the stimulus screen. While the Tobii eye-tracker could detect the monkeys’ eyes and track where they fixated on the screen, experimenters observed many instances when the monkeys were behaviorally looking toward the stimulus screen, yet the eye-tracker did not detect their eyes. Thus, our measures of eye-tracking success with respect to total fixation durations and sampling percent of looking time as reported in the results section are probably an underestimate of a monkeys’ actual looking time. Both from a methodological perspective in working with nonhuman primates, and a software perspective for the sensitivity of eye tracking, there is room for improvement on how to fully capture an unrestrained monkey’s visual attention toward stimuli in an eye-tracking paradigm.

### Conclusion

Eye-tracking methods can be used to understand what primates attend to in their environment, how they process social information, and how to potentially understand differences in social processing as seen in neurodevelopmental disorders such as ASD and schizophrenia. When nonhuman primates have to be sedated or intensely trained in order to participate in a study, the type of monkey able to participate in the study, from age, temperament, or health-wise, become highly restricted. With minimized training involved with the modified transport box method for eye-tracking, it is possible to expand the possibilities for what kind of monkeys can participate in eye-tracking studies. When more monkeys are able to participate, we can increase our generalizability and capture more of the natural variation in their responses which provides more translational value to understanding highly heterogeneous human responses to visual attention and social processing. Furthermore, without sedation, the costs of research can be devoted to increasing sample size for participants rather than the veterinary costs associated with sedation. Non-invasive and unrestrained research methods such as our modified transport box for eye tracking can require less research effort from researchers and animal subjects alike yet still provide meaningful data and positively contribute to expanding our knowledge of social neuroscience.

## Data Availability

The datasets generated for this study are available on request to the corresponding author.

## Author Contributions

MB, KB, TM, and SF conceptualized the study. TM, CH, and MB developed novel methods for collecting eye tracking data in experimentally naive nonhuman primates. TM, SF, AL, MP, and CH developed the methods and oversaw data acquisition. AR, TM, SF, and AL analyzed the data and AR carried out the statistical analyses. SF and TM contributed equally to the work. AR wrote the initial draft of the manuscript and all authors contributed to manuscript revisions. MB and KB jointly funded the research and contributed equally to the work.

## Conflict of Interest Statement

TM contributed to the work while training as a postdoctoral fellow at UC Davis and is currently employed by Sumitomo Dainippon Pharma Co., Ltd, Osaka, Japan. The remaining authors declare that the research was conducted in the absence of any commercial or financial relationships that could be construed as a potential conflict of interest.

## References

[B1] Alvarado M. C. Murphy K. L. Baxter M. G. (2017). Visual recognition memory is impaired in rhesus monkeys repeatedly exposed to sevoflurane in infancy. *Br. J. Anaesthes.* 119 517–523. 10.1093/bja/aew473 28575197 PMC6172969

[B2] Amemori S. Amemori K. I. Cantor M. L. Graybiel A. M. (2015). A non-invasive head-holding device for chronic neural recordings in awake behaving monkeys. *J. Neurosc. Methods* 240 154–160. 10.1016/j.jneumeth.2014.11.006 25448381 PMC4276504

[B3] Bagshaw M. H. Mackworth N. H. Pribram K. H. (1970). Method for recording and analyzing visual fixations in the unrestrained monkey. *Percep. Motor Skills* 31 219–222. 10.2466/pms.1970.31.1.219 4989335

[B4] Bauman M. D. Iosif A. M. Smith S. E. Bregere C. Amaral D. G. Patterson P. H. (2014). Activation of the maternal immune system during pregnancy alters behavioral development of rhesus monkey offspring. *Biol. Psychiatry* 75 332–341. 10.1016/j.biopsych.2013.06.025 24011823 PMC6782053

[B5] Birmingham E. Kingstone A. (2009). Human social attention: A new look at past, present, and future investigations. *Ann. N. Y. Acad. Sci.* 1156 118–140. 10.1111/j.1749-6632.2009.04468.x 19338506

[B6] Black M. H. Chen N. T. M. Iyer K. K. Lipp O. V. Bolte S. Falkmer M. (2017). Mechanisms of facial emotion recognition in autism spectrum disorders: insights from eye tracking and electroencephalography. *Neurosci. Biobehav. Rev.* 80 488–515. 10.1016/j.neubiorev.2017.06.016 28698082

[B7] Chang S. W. C. Brent L. J. N. Adams G. K. Klein J. T. Pearson J. M. Watson K. K. (2013). Neuroethology of primate social behavior. *PNAS* 110 10387–10394. 10.1073/pnas.1301213110 23754410 PMC3690617

[B8] Dahl C. D. Wallraven C. Bulthoff H. H. Logothetis N. K. (2009). Humans and macaques employ similar face-processing strategies. *Curr. Biol.* 19 509–513. 10.1016/j.cub.2009.01.061 19249210

[B9] De Luna P. Mohamed Mustafar M. F. Rainer G. (2014). A MATLAB-based eye tracking control system using non-invasive helmet head restraint in the macaque. *J. Neurosci. Methods* 235 41–50. 10.1016/j.jneumeth.2014.05.033 24979728

[B10] Di Giorgio E. Meary D. Pascalis O. Simion F. (2013). The face perception system becomes species-specific at 3 months: an eye-tracking study. *Int. J. Behav. Dev.* 37 95–99. 10.1177/0165025412465362

[B11] Drucker C. B. Carlson M. L. Toda K. DeWind N. K. Platt M. L. (2015). Non-invasive primate head restraint using thermoplastic masks. *J. Neurosci. Methods* 253 90–100. 10.1016/j.jneumeth.2015.06.013 26112334 PMC4560600

[B12] Duchowski A. T. (2017). *Eye Tracking Methodology: Theory and Practice*. London: Springer 10.1007/978-3-319-57883-5

[B13] Fairhall S. J. Dickson C. A. Scott L. Pearce P. C. (2006). A non-invasive method for studying an index of pupil diameter and visual performance in the rhesus monkey. *J. Med. Primatol.* 35 67–77. 10.1111/j.1600-0684.2005.00135 16556293

[B14] Farah M. J. Wilson K. D. Drain M. Tanaka J. N. (1998). What is “special” about face perception? *Psychol. Rev.* 105 482–498. 9697428 10.1037/0033-295x.105.3.482

[B15] Frazier T. W. Strauss M. Klingemier E. W. Zetzer E. E. Hardan A. Y. Eng C. (2017). A meta-analysis of gaze differences to social and nonsocial information between individuals with and without autism. *J. Am. Acad. Child Adolesc. Psychiatry* 56 546–555. 10.1016/j.jaac.2017.05.005 28647006 PMC5578719

[B16] Goren C. C. Sarty M. Wu P. Y. K. (1975). Visual following and pattern discrimination of face-like stimuli by newborn infants. *Pediatrics* 56 544–549. 1165958

[B17] Gothard K. M. Brooks K. N. Peterson M. A. (2009). Multiple perceptual strategies used by macaque monkeys for face recognition. *Anim. Cogn.* 12 155–167. 10.1007/s10071-008-0179-7 18787848

[B18] Gothard K. M. Erickson C. A. Amaral D. G. (2004). How do rhesus monkeys (*Macaca mulatta*) scan faces in a visual paired comparison task? *Anim. Cogn.* 7 25–36. 10.1007/s10071-003-0179-6 14745584

[B19] Hirata S. Fuwa K. Sugama K. Kusunoki K. Fujita S. (2010). Facial perception of conspecifics: chimpanzees (*Pan troglodytes*) preferentially attend to proper orientation and open eyes. *Anim. Cogn.* 13 679–688. 10.1007/s10071-010-0316-y 20213188

[B20] Howard L. H. Festa C. Lonsdorf E. V. (2018). Through their eyes: the influence of social models on attention and memory in capuchin monkeys (*Sapajus apella*). *J. Comp. Psychol.* 132 210–219. 10.1037/com0000111 29517249

[B21] Howard L. H. Wagner K. E. Woodward A. L. Ross S. R. Hopper L. M. (2017). Social models enhance apes’ memory for novel events. *Sci. Rep.* 7:40926. 10.1038/srep40926 28106098 PMC5247682

[B22] Imafuku M. Kawai M. Niwa F. Shinya Y. Inagawa M. Myowa-Yamakoshi M. (2017). Preference for dyanmic human images and gaze-following abilities in preterm infants at 6 and 12 months of age: an eye-tracking study. *Infancy* 22 223–239. 10.1111/infa.12144 33158339

[B23] Ioannidou F. Hermens F. Hodgson T. L. (2017). Mind your step: the effects of mobile phone use on gaze behavior in stair climbing. *J. Technol. Behav. Sci.* 2 109–120. 10.1007/s41347-017-0022-6 29387779 PMC5770487

[B24] Judge S. J. Richmond B. J. Chu F. C. (1980). Implantation of magnetic search coils for measurement of eye position: an improved method. *Vis. Res.* 20 535–538. 10.1016/0042-6989(80)90128-5 6776685

[B25] Kano F. Call J. Tomonaga M. (2012). Face and eye scanning in gorillas (*Gorilla gorilla*), orangutans (*Pongo abelii*), and humans (*Homo sapiens*): unique eye-viewing patterns in humans among hominids. *J. Comp. Psychol.* 126 388–398. 10.1037/a0029615 22946925

[B26] Kano F. Shepherd S. V. Hirata S. Call J. (2018). Primate social attention: species differences and effects of individual experience in humans, great apes, and macaques. *Plos One* 13:e0193283. 10.1371/journal.pone.0193283 29474416 PMC5825077

[B27] Kano F. Tomonaga M. (2009). How chimpanzees look at pictures: a comparative eye-tracking study. *Proc. R. Soc. B-Biol. Sci.* 276 1949–1955. 10.1098/rspb.2008.1811 19324790 PMC2677242

[B28] Keating C. F. Keating E. G. (1982). Visual scan patterns of rhesus monkeys viewing faces. *Perception* 11 211–219. 10.1068/p110211 7155774

[B29] Khachatryan H. Rihn A. L. Campbell B. Yue C. Y. Hall C. Behe B. (2017). Visual attention to eco-labels predicts consumer preferences for pollinator friendly plants. *Sustainability* 9:1743 10.3390/su9101743

[B30] Landi S. M. Freiwald W. A. (2017). Two areas for familiar face recognition in the primate brain. *Science* 357 591–595. 10.1126/science.aan1139 28798130 PMC5612776

[B31] Leonard T. K. Blumenthal G. Gothard K. M. Hoffman K. L. (2012). How macaques view familiarity and gaze in conspecific faces. *Behav. Neurosci.* 126 781–791. 10.1037/a0030348 23067381

[B32] Lewkowicz D. J. Hansen-Tift A. M. (2012). Infants deploy selective attention to the mouth of a talking face when learning speech. *PNAS* 109 1431–1436. 10.1073/pnas.1114783109 22307596 PMC3277111

[B33] Loughland C. M. Williams L. M. Gordon E. (2002). Schizophrenia and affective disorder show different visual scanning behavior for faces: a trait versus state-based distinction? *Biol. Psychiatry* 52 338–348. 10.1016/S0006-3223(02)01356-2 12208641

[B34] Machado C. J. Bliss-Moreau E. Platt M. L. Amaral D. G. (2011). Social and nonsocial content differentially modulates visual attention and autonomic arousal in rhesus macaques. *Plos One* 6:10. 10.1371/journal.pone.0026598 22046313 PMC3202553

[B35] Machado C. J. Nelson E. E. (2011). Eye-tracking with nonhuman primates is now more accessible than ever before. *Am. J. Primatol.* 73 562–569. 10.1002/ajp.20928 21319204 PMC3084536

[B36] Machado C. J. Whitaker A. M. Smith S. E. P. Patterson P. H. Bauman M. D. (2015). Maternal immune activation in nonhuman primates alters social attention in juvenile offspring. *Biol. Psychiatry* 77 823–832. 10.1016/j.biopsych.2014.07.035 25442006 PMC7010413

[B37] Marwick K. Hall J. (2008). Social cognition in schizophrenia: a review of face processing. *Br. Med. Bull.* 88 43–58. 10.1093/bmb/ldn035 18812413

[B38] Millan M. J. Bales K. L. (2013). Towards improved animal models for evaluating social cognition and its disruption in schizophrenia: the CNTRICS initiative. *Neurosci. Biobehav. Rev.* 37(9 Pt B), 2166–2180. 10.1016/j.neubiorev.2013.09.012 24090822

[B39] Mitchell J. F. Reynolds J. H. Miller C. T. (2014). Active vision in marmosets: a model system for visual neuroscience. *J. Neurosci.* 34 1183–1194. 10.1523/jneurosci.3899-13.2014 24453311 PMC3898283

[B40] Mosher C. P. Zimmerman P. E. Gothard K. M. (2011). Videos of conspecifics elicit interactive looking patterns and facial expressions in monkeys. *Behav. Neurosci.* 125 639–652. 10.1037/a0024264 21688888 PMC4184141

[B41] Muschinski J. Feczko E. Brooks J. M. Collantes M. Heitz T. R. Parr L. A. (2016). The development of visual preferences for direct versus averted gaze faces in infant macaques (*Macaca mulatta*). *Dev. Psychobiol.* 58 926–936. 10.1002/dev.21421 27195755

[B42] Nahm F. K. D. Perret A. Amaral D. G. Albright T. D. (1997). How do monkeys look at faces? *J. Cogn. Neurosci.* 9 611–623. 10.1162/jocn.1997.9.5.611 23965120

[B43] Papageorgiou K. A. Smith T. J. Wu R. Johnson M. H. Kirkham N. Z. Ronald A. (2014). Individual differences in infant fixation duration relate to attention and behavioral control in childhood. *Psychol. Sci.* 25 1371–1379. 10.1177/0956797614531295 24815614

[B44] Papagiannopoulou E. A. Chitty K. M. Hermens D. F. Hickie I. B. Lagopoulos J. (2014). A systematic review and meta-analysis of eye-tracking studies in children with autism spectrum disorders. *Soc. Neurosci.* 9 610–632. 10.1080/17470919.2014.934966 24988218

[B45] Paukner A. Bower S. Simpson E. A. Suomi S. J. (2013). Sensitivity to first-order relations of facial elements in infant rhesus macaques. *Inf. Child Dev.* 22 320–330. 10.1002/icd.1793 23997657 PMC3753110

[B46] Phillips K. A. Bales K. L. Capitanio J. P. Conley A. Czoty P. W. Hart B. A. (2014). Why primate models matter. *Am. J. Primatol.* 76 801–827. 10.1002/ajp.22281 24723482 PMC4145602

[B47] Pritsch C. Telkemeyer S. Muhlenbeck C. Liebal K. (2017). Perception of facial expressions reveals selective affect-biased attention in humans and orangutans. *Sci. Rep.* 7:7782. 10.1038/s41598-017-07563-4 28798378 PMC5552869

[B48] Putnam P. T. Roman J. M. Zimmerman P. E. Gothard K. M. (2016). Oxytocin enhances gaze-following responses to videos of natural social behavior in adult male rhesus monkeys. *Psychoneuroendocrinology* 72 47–53. 10.1016/j.psyneuen.2016.05.016 27343726 PMC5226068

[B49] Roy A. Shepherd S. V. Platt M. L. (2014). Reversible inactivation of pSTS suppresses social gaze following in the macaque (*Macaca mulatta*). *Soc. Cogn. Affect. Neurosci.* 9 209–217. 10.1093/scan/nss123 23171617 PMC3907927

[B50] Simpson E. A. Jakobsen K. V. Damon F. Suomi S. J. Ferrari P. F. Paukner A. (2017). Face detection and the development of own-species bias in infant macaques. *Child Dev.* 88 103–113. 10.1111/cdev.12565 27223687 PMC5123966

[B51] Sliwa J. Duhamel J.-R. Pascalis O. Wirth S. (2011). Spontaneous voice–face identity matching by rhesus monkeys for familiar conspecifics and humans. *PNAS* 108 1735–1740. 10.1073/pnas.1008169108 21220340 PMC3029706

[B52] Venker C. E. Kover S. T. (2015). An open conversation on using eye-gaze methods in studies of neurodevelopmental disorders. *J. Speech Lang. Hear. Res.* 58 1719–1732. 10.1044/2015_Jslhr-L-14-0304 26363412 PMC4987028

[B53] Williams C. C. Henderson J. M. (2007). The face inversion effect is not a consequence of aberrant eye movements. *Mem. Cogn.* 35 1977–1985. 10.3758/Bf03192930 18265613

[B54] Wilson C. R. E. Buckley M. J. Gaffan D. (2010). Degraded transfer of memories between the visual hemifields in normal macaques revealed by a novel infrared eyetracking method without head fixation. *Neuropsychologia* 48 1376–1384. 10.1016/j.neuropsychologia.2010.01.003 20079363

