# Supplementary material for: Non-invasive Eye Tracking Methods for New World and Old World Monkeys
Source: Front Behav Neurosci. 2019 Mar 5;13:39. doi: 10.3389/fnbeh.2019.00039 (PMC6412371; doi:10.3389/fnbeh.2019.00039)
Supplement: Supplementary file 1 [file Data_Sheet_1.pdf]

# Supplementary Material

## Macaque Stimulus Sets

### 1. Facial Expressions

Forty images (approximately 550 x 500 pixels each) of unfamiliar adult macaque facial expressions were donated by Dr. Machado. These facial expressions included neutral (n=10), lipsmack (n=10), fear grimace (n=10), and open-mouth threat (n=10). Three different stimulus subsets were prepared, and each set consisted of 12 images, 3 of each facial expression, in a randomized order. Each image was presented for 15 seconds and was preceded and followed by a blank screen for 10 seconds. The three subsets of facial expressions were viewed twice by each monkey across the six test sessions.

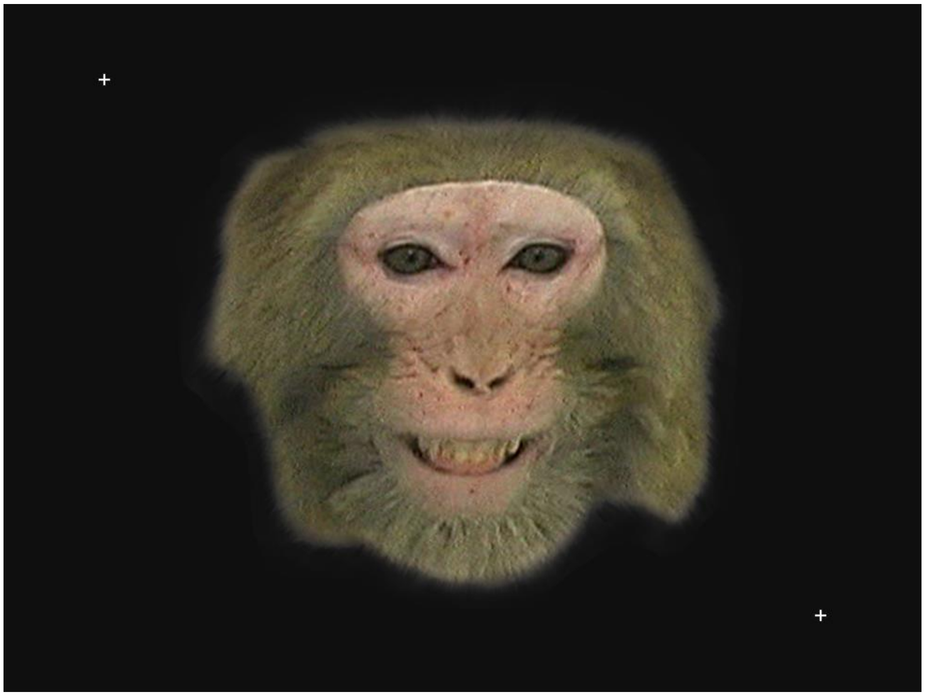

Figure 1. One of the facial expression stimuli presented to monkeys for 15 seconds during the Facial Expressions stimuli presentation.

**2. Social vs. Nature video clips**

Video clips were donated by Dr. Machado and described further in Machado et al. (2014). In each testing session, there were 10 stimuli (all 1080 x 545 pixels) shown for 30 seconds each with a black screen shown for 10 seconds in between stimuli. Five of the stimuli were videos of unfamiliar rhesus macaques. Of the five stimuli, three of them showed rhesus macaques foraging or engaging in social behaviors such as aggression, grooming, playing, and mounting. The other two showed a single monkey directing facial expressions at the camera such as aggression, submission, or neutral. The five nature videos consisted of one stimulus from each category: landscapes, land mammals, marine mammals, birds, or insects or other invertebrates. None of the nature videos showed humans, other nonhuman primate species, or other natural predators of macaques (i.e., snakes, predatory cats, or large reptiles). The ten stimuli were randomly presented in a testing session. We created six stimulus sets of novel social and nature stimuli and the monkeys viewed each stimulus set once across the six time points.

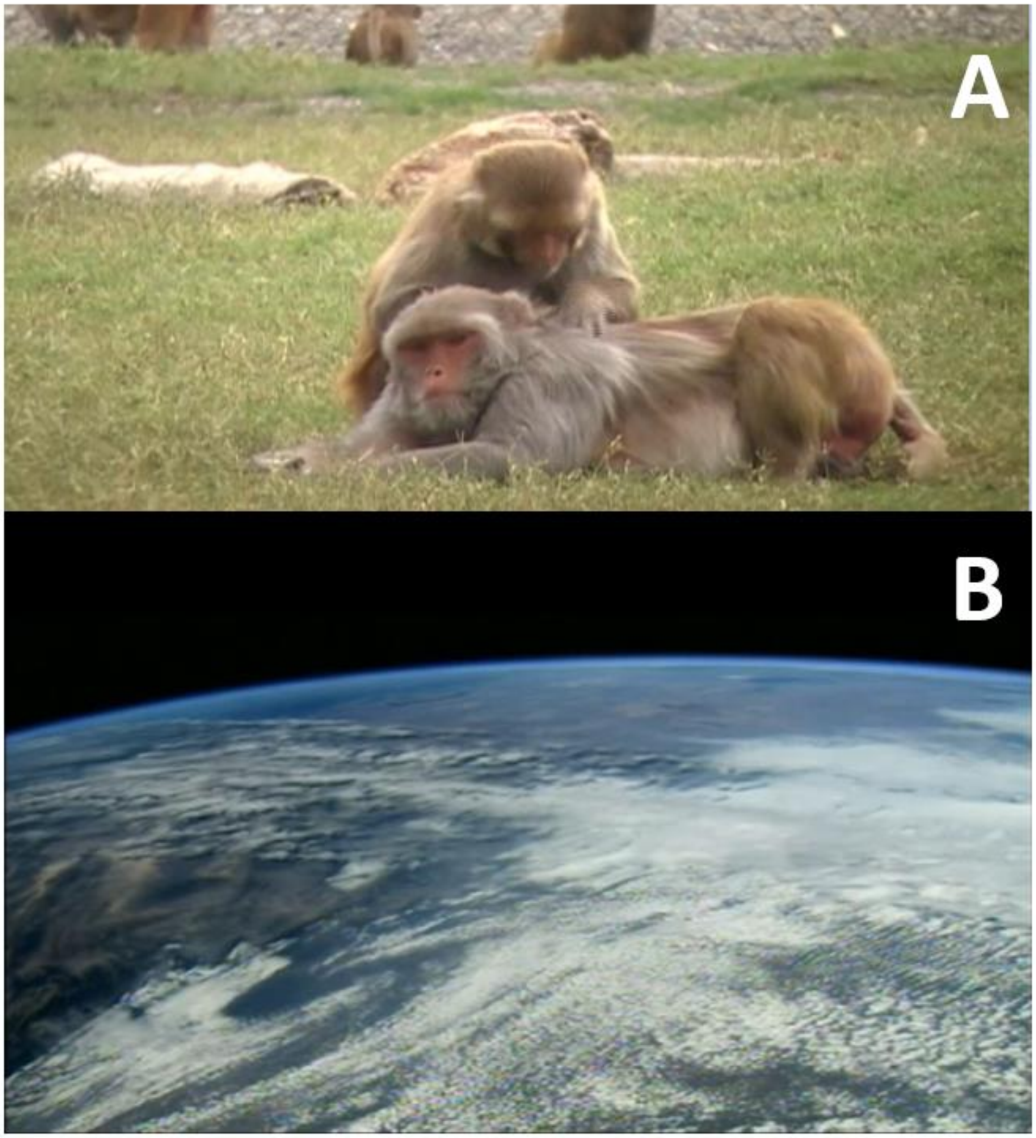


Figure 2. Still frames of one of the rhesus macaque stimuli (a) and nature stimuli (b) presented to the monkeys during the Social versus Nature stimuli set.

**3. Social vs. Abstract video clips**

In this stimulus set, donated by Dr. Annika Paukner, primate social stimuli (approximately 560 x 320 pixels) were presented side-by-side with an abstract shape (70 x 60 pixels) that continuously moved across the screen. The primate social stimuli were either of

monkeys engaging in social behavior such as grooming or of humans engaging in a conversation. Each stimulus was presented for 36 seconds with no blank screen in between stimulus presentations. There were four pairs of nonsocial and social stimuli, with two monkey social and two human social clips presented with the same abstract shape. The stimulus set of four pairs was presented in all 6 data collection sessions. The social scene was presented on either the right or left side of the screen, and a nonsocial scene (abstract shapes continuously moving across the screen) was presented on the other side of the screen. The presentation side of the social and nonsocial scenes were balanced across the four clips.

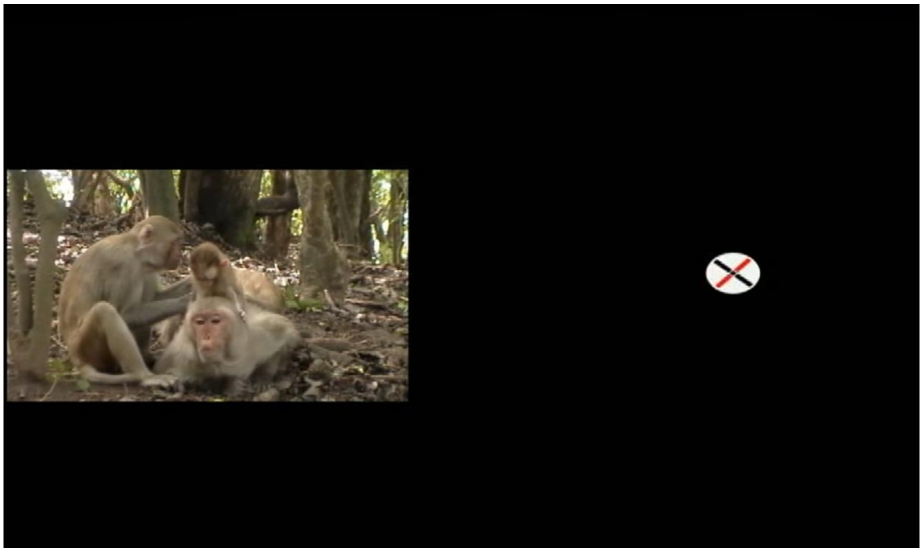

Figure 3. A still frame of one of the monkey vs. abstract shape stimuli displayed to the monkeys during the Social vs. Abstract stimuli set.

### 4. Mother-infant interaction video clip

We presented the monkeys with a continuous 70-second video clip of a mother-infant interaction in a naturalistic setting (1630 x 1080 pixels). The monkeys viewed the video clip in all 6 sessions.

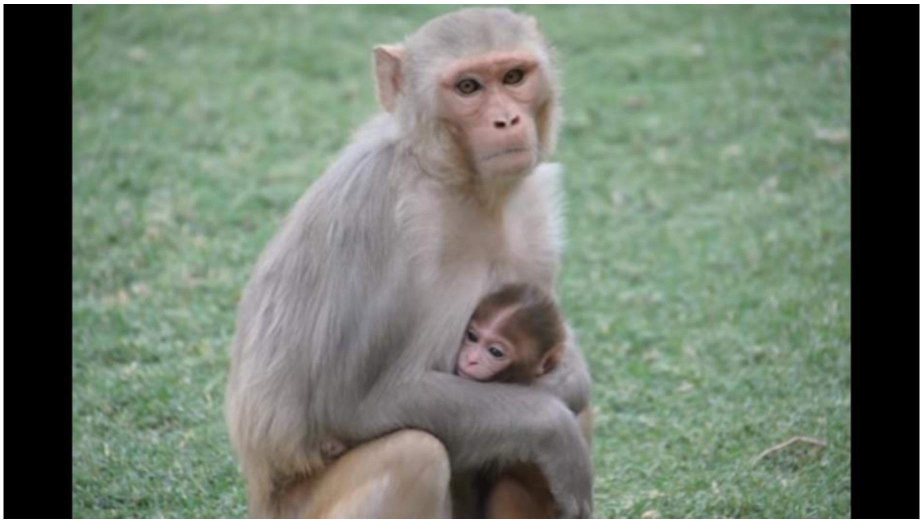

Figure 4. A still frame from the mother-infant interaction presented during the Mother-infant interaction video clip.

**Titi Monkey Stimulus Sets**

**1. Photo stimuli**

Photographs of titi monkeys in their home cages were presented as a single image on the screen, shown for 10 seconds, with a three-second black screen in between stimuli. The photos varied in size from full screen through narrower photos with a range from 959 x 716 pixels through 484 x 720 pixels. The stimulus monkeys shown were other titi monkeys in the colony but unfamiliar to the titi monkeys being tested. Eight of the stimuli were most or full body images of titi monkeys and two were a close-up of the face, with 11 total photos shown. One photo had a food hopper (nonsocial image) in the foreground and a titi monkey in the background. The stimuli were randomly presented in a testing session.

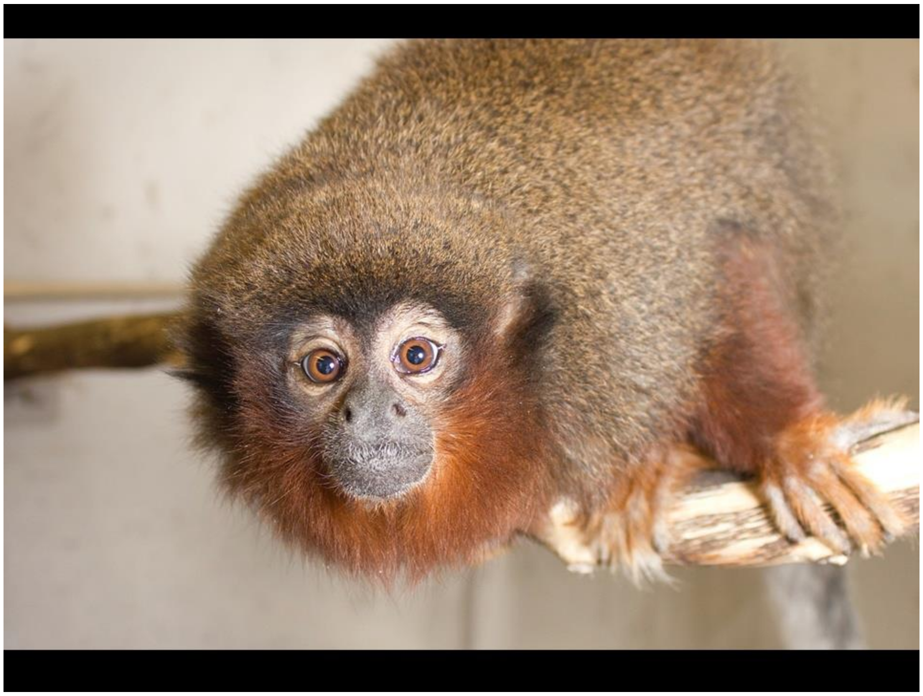

Figure 5. One of the photos of a titi monkey presented to the monkeys during the Photo stimuli set.

**2. Video Clips**

Six videos of titi monkeys in their home cages were presented for 10 seconds each with a three-second break in between. The videos varied in size from full screen through narrower videos with a range from 1440 x 810 pixels through 1440 x 810 pixels. The videos consisted of titi monkeys sitting in proximity to each other with audio of vocalizations. The stimulus monkeys were from the same research colony as the subject monkeys but were unfamiliar to the subject monkeys. The presentation order for the six videos was randomized for each session.

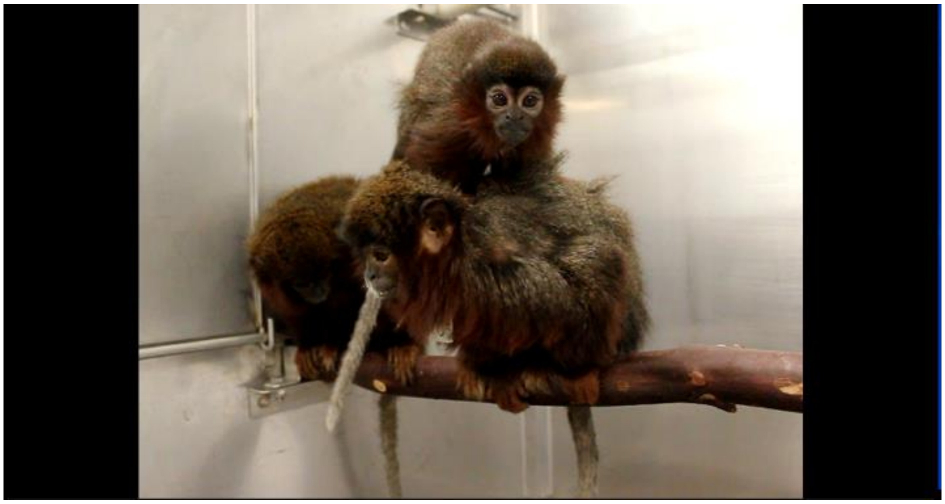

Figure 6. A still frame from one video presented during the titi Video Clips stimuli presentation.
